# Post-acute metabolic changes and risk of new-onset diabetes following COVID-19: a systematic review and meta-analysis

**DOI:** 10.3389/fendo.2026.1835180

**Published:** 2026-05-21

**Authors:** Bukola Lawal, Shuaibu Saidu Musa, May Soe Thu, Thunnicha Ondee, Oranut Chatsirisakul, Krit Pongpirul

**Affiliations:** 1 Graduate Program in Medical Sciences, Faculty of Medicine, Chulalongkorn University, Bangkok, Thailand; 2 Graduate Program in Clinical Sciences, Faculty of Medicine, Chulalongkorn University, Bangkok, Thailand; 3 Center of Excellence in Immunology and Immune-Mediated Diseases, Department of Microbiology, Faculty of Medicine, Chulalongkorn University, Bangkok, Thailand; 4 Thai Red Cross Society, Bangkok, Thailand; 5 Faculty of Medicine, Chulalongkorn University, Bangkok, Thailand; 6 Clinical Research Center, Bumrungrad International Hospital, Bangkok, Thailand; 7 Center of Excellence in Preventive and Integrative Medicine (CE-PIM), Faculty of Medicine, Chulalongkorn University, Bangkok, Thailand; 8 Department of Infection Biology & Microbiomes, Faculty of Health and Life Sciences, University of Liverpool, Liverpool, United Kingdom

**Keywords:** COVID-19, long COVID, post-acute sequelae of SARS-CoV-2, new-onset diabetes, insulin resistance, HbA1c, metabolic dysregulation, meta-analysis

## Abstract

**Background:**

COVID-19 has been associated with persistent metabolic disturbances; however, the magnitude, consistency, and underlying mechanisms of post-infection alterations in glucose regulation remain incompletely characterized.

**Methods:**

We conducted a systematic review and meta-analysis in accordance with PRISMA guidelines. PubMed and Embase were searched on December 18, 2024, for studies published from 2020 onward. Eligible studies included observational cohort and cross-sectional designs assessing metabolic outcomes at least three months after recovery from COVID-19.

**Results:**

Sixteen studies met inclusion criteria. Pooled analysis suggested a 41% increased risk of new-onset diabetes among COVID-19 survivors compared with non-infected individuals (RR 1.41, 95% CI: 1.38–1.44); however, this estimate was predominantly driven by a single large-scale study. Quantitative synthesis demonstrated higher HbA1c (SMD 1.44, 95% CI: 0.36–2.52) and Homeostatic Model Assessment of Insulin Resistance (HOMA-IR) (SMD 0.96, 95% CI: 0.33–1.58), consistent with impaired glycemic control and increased insulin resistance. In contrast, fasting blood glucose (FBG) findings were inconsistent and highly heterogeneous (SMD 0.77, 95% CI: −0.40–1.94). Substantial heterogeneity was observed across outcomes.

**Conclusion:**

COVID-19 may be associated with an increased risk of incident diabetes and persistent metabolic dysregulation. However, the limited number of studies contributing to pooled risk estimates and the influence of large registry-based data warrant cautious interpretation. These findings support consideration of metabolic monitoring and longitudinal follow-up in post-COVID care, particularly among individuals at elevated cardiometabolic risk.

**Systematic review registration:**

https://www.crd.york.ac.uk/prospero/, identifier CRD42025630971.

## Introduction

Severe Acute Respiratory Syndrome Coronavirus 2 (SARS-CoV-2), the causative agent of COVID-19, has exerted substantial effects on global health systems and economies since its emergence (1). Although COVID-19 is primarily a respiratory illness, its clinical impact extends beyond the lungs, affecting multiple organ systems, including the endocrine pancreas. While most individuals experience mild disease, severe outcomes are more common among older adults, immunocompromised individuals, and those with underlying comorbidities (2).

In addition to acute manifestations, a proportion of individuals experience persistent or delayed complications following recovery. This condition, commonly referred to as “long COVID” or post-acute sequelae of SARS-CoV-2 infection (PASC), is characterized by symptoms lasting at least three months after the initial infection and may affect individuals across all age groups, including children (3, 4). These long-term sequelae increasingly include metabolic disturbances, highlighting the systemic nature of SARS-CoV-2 infection.

Recent epidemiological data continue to demonstrate ongoing transmission and clinical burden of COVID-19. Reports from the Centers for Disease Control and Prevention (CDC) in 2025 indicated an increase in weekly hospital admissions in the United States from approximately 6,500 in early August to nearly 8,000 by the end of the month. Although overall mortality remained relatively stable, a substantial proportion of severe outcomes, including intensive care unit admissions and deaths, occurred among older adults, underscoring the continued relevance of SARS-CoV-2 as a public health concern (5, 6).

The mechanisms underlying post-COVID-19 complications are not yet fully elucidated, but accumulating evidence suggests that disease severity and host factors during the acute phase may influence the persistence of long-term sequelae (7). Among these, disturbances in glucose metabolism have gained increasing attention. Emerging evidence suggests that individuals without prior diabetes may develop new-onset type 2 diabetes mellitus (T2DM) following COVID-19 infection (8). Large-scale cohort studies and meta-analyses have reported an elevated risk of incident diabetes after SARS-CoV-2 infection, although the magnitude and consistency of this association vary across populations and study designs (9–11).

The relationship between COVID-19 infection and diabetes appears to be bidirectional (12–14). From a pathophysiological perspective, SARS-CoV-2 gains cellular entry through angiotensin-converting enzyme 2 (ACE2) receptors, expressed in pancreatic tissue, raising the possibility of direct viral effects on pancreatic islet cells and impaired β-cell function. In addition, systemic immune activation and inflammatory responses, characterized by elevated cytokines such as interleukin-6 (IL-6), tumor necrosis factor-α (TNF-α), and interferon-gamma (IFN-γ), have been implicated in the disruption of insulin signaling pathways and the development of insulin resistance (15, 16). Longitudinal evidence further suggests that post-COVID syndrome may be associated with persistent insulin resistance over time, even among individuals with low baseline metabolic risk, supporting a role for chronic inflammation in driving metabolic dysfunction (17). These processes may extend beyond the acute phase and contribute to sustained metabolic dysregulation.

More broadly, these observations align with the emerging conceptualization of diabetes as an immunometabolic disorder, in which metabolic and immune pathways are tightly interconnected and may be amplified following viral infections (18). This framework provides a basis for understanding the heterogeneity and persistence of metabolic complications observed after COVID-19.

Despite a growing body of literature, important gaps remain. Many studies focus primarily on incident diabetes without evaluating underlying metabolic changes, while heterogeneity in study design, populations, and timing of assessment limits comparability across findings. In addition, the extent to which large registry-based datasets influence pooled estimates has not been consistently examined.

To address these gaps, we conducted a systematic review and meta-analysis to (i) quantify the risk of new-onset diabetes following COVID-19 infection and (ii) evaluate changes in key metabolic parameters, including fasting blood glucose (FBG), fasting insulin, Homeostatic Model Assessment of Insulin Resistance (HOMA-IR), and hemoglobin A1c (HbA1c), assessed at least three months after infection. By integrating epidemiological and biomarker-based evidence and incorporating sensitivity analyses, this study aims to provide a more comprehensive and critically appraised assessment of post-COVID metabolic dysfunction and its potential clinical implications.

## Materials and method

### Protocol

This systematic review and meta-analysis was conducted in accordance with the Preferred Reporting Items for Systematic Reviews and Meta-Analyses (PRISMA) 2020 guidelines (19). The study protocol was prospectively registered in the PROSPERO database (registration number: CRD42025630971).

The review was structured according to the PICOS framework: Population (individuals recovering from COVID-19, with or without pre-existing dysglycemia), Exposure (SARS-CoV-2 infection), Comparator (non-infected individuals or pre-infection baseline), Outcomes (new-onset diabetes, HbA1c, fasting blood glucose, HOMA-IR), and Study design (observational cohort and cross-sectional studies) (19).

### Literature search and study selection

On December 18, 2024, a systematic search of published articles was performed in PubMed (pubmed.ncbi.nlm.nih.gov) and Embase (www.embase.com) to identify English-language studies reporting metabolic changes following COVID-19. The search strategy combined controlled vocabulary (MeSH/Emtree terms) and free-text keywords related to diabetes and glucose metabolism (e.g., “diabetes mellitus,” “blood glucose,” “insulin resistance”) with terms describing post-COVID conditions (e.g., “post-acute COVID-19 syndrome,” “long COVID,” “post-COVID,” “SARS-CoV-2 survivors”). Boolean operators were applied to combine these concepts, and the search was restricted to studies published from 2020 onwards (see **Supplementary S1** for full search strategy). Search results were exported and deduplicated prior to screening.

The inclusion and exclusion criteria were defined using the PICOS framework. We included studies assessing glucose metabolism or related metabolic parameters in individuals recovering from COVID-19, with or without pre-existing dysglycemia, at least three months after infection. Eligible outcomes included incident diabetes or changes in glycemic markers (e.g., HbA1c, fasting blood glucose, HOMA-IR).

We excluded studies evaluating participants during the acute infection period (<3 months post-infection), as well as those without relevant metabolic outcomes. Reviews, case reports, and other non-original studies were excluded. Both observational cohort and cross-sectional studies meeting predefined inclusion criteria were retained.

After removing duplicates, three investigators independently screened titles and abstracts. Full texts of potentially eligible studies were subsequently reviewed for final inclusion. Discrepancies were resolved through discussion and consensus.

### Outcomes

The primary outcome was the occurrence of new-onset diabetes following COVID-19 infection, as defined according to the diagnostic criteria reported in individual studies. Secondary outcomes included changes in metabolic parameters at least three months after infection, including FBG, HbA1c, HOMA-IR, and fasting insulin levels.

### Definitions of metabolic parameters

FBG: Concentration of glucose in the blood after a minimum of 8 hours of fasting, reported in mmol/L or mg/dL. It is a standard diagnostic marker for diabetes and impaired fasting glucose. A value ≥7.0 mmol/L (126 mg/dL) is consistent with diabetes mellitus according to established clinical criteria.

HbA1c: Reflects average blood glucose levels over the preceding 2–3 months and is expressed as a percentage (or mmol/mol). An HbA1c ≥6.5% (48 mmol/mol) is indicative of diabetes, while intermediate values (5.7–6.4%) suggest prediabetes.

HOMA-IR: An index used to estimate insulin resistance, calculated as: HOMA-IR = [fasting insulin (μU/mL) × FBG (mmol/L)]/22.5. Higher values indicate greater insulin resistance; however, no universal cutoff exists, and thresholds may vary across populations and study settings.

### Data extraction and management

Data extraction was performed using a standardized form to collect study characteristics, including authors, publication year, study period, study design, trial registration (if available), and country of origin. Participant characteristics were recorded, including sample size, age, sex, and relevant clinical conditions).

Extracted outcomes included metabolic parameters of interest, namely FBG, HbA1C, HOMA-IR, fasting insulin levels. Pre-specified variables such as short-chain fatty acids (SCFAs) and other metabolomic markers were also sought but were not consistently reported across studies and were therefore not included in the quantitative synthesis.

Two authors independently performed data extraction. Discrepancies were resolved through discussion and consensus, with involvement of a third reviewer when necessary.

### Risk of bias assessment

Risk of bias was independently assessed by two reviewers, with disagreements resolved through discussion and consensus, and involvement of a third reviewer when necessary (20).

For cohort studies (prospective, retrospective, and registry-based), the Newcastle-Ottawa Scale (NOS) was used to evaluate methodological quality (21). This tool assesses three domains: selection of study groups (0–4 points), comparability of groups (0–2 points), and outcome assessment (0–3 points), with a maximum score of 9. Studies scoring ≥7 were considered to be of high quality (low risk of bias).

For cross-sectional studies, the Joanna Briggs Institute (JBI) Critical Appraisal Checklist for Analytical Cross-Sectional Studies was applied (22). This 8-item tool evaluates study design, exposure measurement (COVID-19 diagnosis), outcome assessment (diabetes and metabolic parameters), and control of confounding. Studies were categorized as low, moderate, or high risk of bias based on the number of “Yes” responses (7-8, 4-6, and 0–3 items, respectively).

### Statistical analysis

Descriptive statistics, including means and standard deviations (SD), were used to summarize study-level and participant characteristics.

Meta-analyses were conducted using a random-effects model according to the DerSimonian and Laird method (20) to account for anticipated between-study heterogeneity arising from differences in study design, participant characteristics, and COVID-19 severity. Effect sizes were expressed as pooled relative risks (RR) for dichotomous outcomes and standardized mean differences (SMD) for continuous outcomes, each with corresponding 95% confidence intervals (CIs). Forest plots were generated to visualize individual study estimates and pooled effects.

Statistical heterogeneity was assessed using the *I²* statistic (37) and interpreted as low (0–25%), moderate (26–50%), or high (>50%). Sensitivity analyses were performed using a leave-one-out approach, whereby individual studies were sequentially excluded to evaluate their influence on pooled estimates and heterogeneity. Publication bias was assessed using funnel plots for outcomes with a sufficient number of studies; however, formal assessment was limited for analyses with fewer studies. All analyses were performed using Review Manager (RevMan) version 5.4. A two-sided *p* < 0.05 was considered statistically significant.

## Results

### Study selection

A total of 4,572 records were identified from electronic databases, including 2,313 from Embase and 2,259 from PubMed. After removal of 702 duplicates, 3,870 unique records remained for screening. Following title and abstract screening, 3,442 records were excluded, leaving 428 articles for full-text assessment.

Of these, 412 studies were excluded for the following reasons: duplicate (n = 1), animal studies (n = 2), wrong setting (n = 227), wrong outcome (n = 6), wrong comparator (n = 2), wrong intervention (n = 125), non-peer reviewed articles (n = 43), and wrong patient population (n = 6). Categories of wrong setting, intervention, and comparator categories were defined *a priori* based on PICOS criteria (e.g., studies not evaluating post-acute COVID-19 populations or lacking appropriate comparison groups). A total of 16 studies met the eligibility criteria and were included in the final analysis. The characteristics of the included studies are summarized in **Table 1**.

**Table 1:** Characteristics of included studies.

Author (year)	Country	Study design	Follow-up	Total (N)	Population/subgroup	Comparator	Age (mean ± Sd)	Diagnostic method	Fbg	Homa-Ir	Hba1c
Stephen (23)	Nigeria	Retrospective cohort	–	512	General population	COVID+ vs control	39.84 vs 40.74	RT-PCR	Pre-DM 27%, DM 7% vs 4%, 2%	–	–
Man (1)	Romania	Prospective cohort	–	143	Long COVID patients	COVID+ vs control	55.05 ± 10.52	RT-PCR	~106 mg/dL	4.08	–
Santos (24)	Brazil	Cross-sectional	12 mo	77	Fatigue vs non-fatigue (post-COVID)	Subgroup comparison	53.7 ± 11.7 vs 56.2 ± 11.8	–	–	–	–
Lui (25)	Hong Kong	Longitudinal cohort	1 y	102	COVID survivors	COVID+ vs control	61.2 ± 8.8 vs 59.8 ± 3.5	RT-PCR	5.06→5.17 mmol/L	1.39→1.47	5.5→5.7%
Xu (26)	USA	Retrospective cohort	5 mo	37,888	Prediabetes cohort	COVID+ vs matched	56.8 vs 57.0	RT-PCR	112.15 ± 26.27 vs 101.27 ± 39.51 mg/dL	–	5.7 ± 0.36 vs 5.8 ± 0.26%
Sharma (27)	USA	Retrospective cohort	12 mo	20,753	T2D patients	COVID+ vs control	61.6 vs 63.7	RT-PCR	–	–	6.7% vs 6.7%
Zisis (28)	USA	Matched cohort	3–12 mo	5,197,096	General population	COVID+ vs matched	44.4 vs 47.5	–	–	–	–
Parimala (29)	India	Cross-sectional	–	30	Post-COVID diabetics vs non-diabetics	Subgroup comparison	–	–	–	–	9.82 ± 3.22 vs 5.83 ± 3.22%
Rathore (30)	India	Prospective cohort	3 mo	100	Pre-diabetes cohort	Developed vs not developed diabetes	–	RT-PCR/antigen	113.33 ± 8.34 vs 101.52 ± 28.88 mg/dL	–	–
Pietrzak (31)	Poland	Cross-sectional	–	3,062	New-onset T1D	Pre vs post COVID period	9.5 ± 4.3 (years)	–	–	–	114.8 ± 28.9 mmol/mol
Shestakova (32)	Russia	Prospective cohort	6–52 wk	194	Post-COVID patients	Severity groups	~58 years	RT-PCR/CT	5.03–8.2 mmol/L	–	~7.7→7.8%
Montefusco (33)	Italy	Observational cohort	2 mo	551	Glycemic subgroups	Hyperglycemic vs normoglycemic	55–67	–	140 vs 90 mg/dL	3.5 vs 1.9	7.0 vs 5.4%
Alberca (34)	Brazil	Prospective cohort	3–6 mo	128	Diabetic vs non-diabetic	Subgroup comparison	60.5 vs 62	RT-PCR	118.5 vs 94 mg/dL	–	6.6 vs 6.0%
Choi (35)	South Korea	Nationwide cohort	2 y	1,392,720	General population	COVID+ vs matched	43.3	–	–	–	–
Goel (36)	India	Historical cohort	6 mo	70	Post-COVID patients	Baseline vs follow-up	52.2 ± 17.4	RT-PCR	~176→160 mg/dL	–	–
Jamwal (2024)	India	Cohort	6 mo	855	New-onset diabetes cohort	Baseline vs follow-up	–	–	142.36→116.5 mg/dL	5.17→3.80	6.5→6.1%

Values are presented as mean ± standard deviation (SD), percentage, or range as reported in the original studies. FBG values are reported in original units (mg/dL or mmol/L), and HbA1c values are reported in % or mmol/mol as provided. “–” indicates data not reported. Arrows (→) indicate change over time within the same cohort.

FBG, fasting blood glucose; HOMA-IR, Homeostatic Model Assessment of Insulin Resistance; HbA1c, glycated hemoglobin; T1D, type 1 diabetes; T2D, type 2 diabetes; NODAC, new-onset diabetes after COVID-19; NODM, new-onset diabetes mellitus; NOPD, new-onset prediabetes; RT-PCR, reverse transcription polymerase chain reaction; CGM, continuous glucose monitoring; COVID+, COVID-19 positive; COVID−, COVID-19 negative; mo, months; y, years.

**Figure 1:**
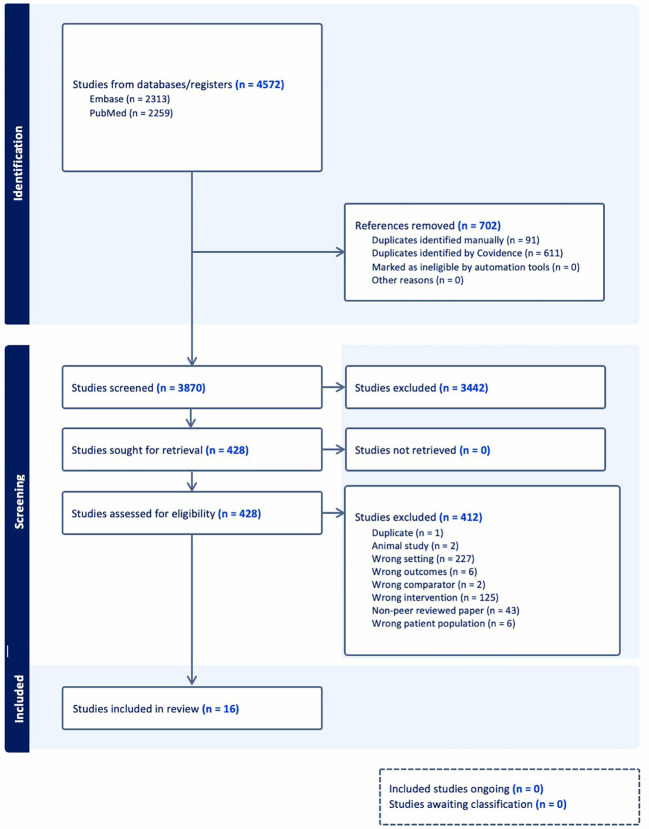
PRISMA flow diagram illustrating the study selection process, including identification, screening, eligibility, and inclusion of studies in the systematic review and meta-analysis.

The included studies were published between 2020 and 2024, with the majority adopting cohort designs. Studies were conducted across diverse geographic regions, including the Americas, Asia, Europe, and Africa. Among the 16 included studies, most evaluated metabolic outcomes following COVID-19 recovery, with 10 reporting quantitative glucose-related biomarkers (e.g., FBG, HbA1c, or HOMA-IR) longitudinally or comparatively. The remaining studies either reported incident diabetes without detailed biomarker data or focused on subgroup or non-metabolic outcomes. The full study selection process is illustrated in the PRISMA flow diagram (**Figure 1**).

### Risk of bias assessment

**Figure 2:**
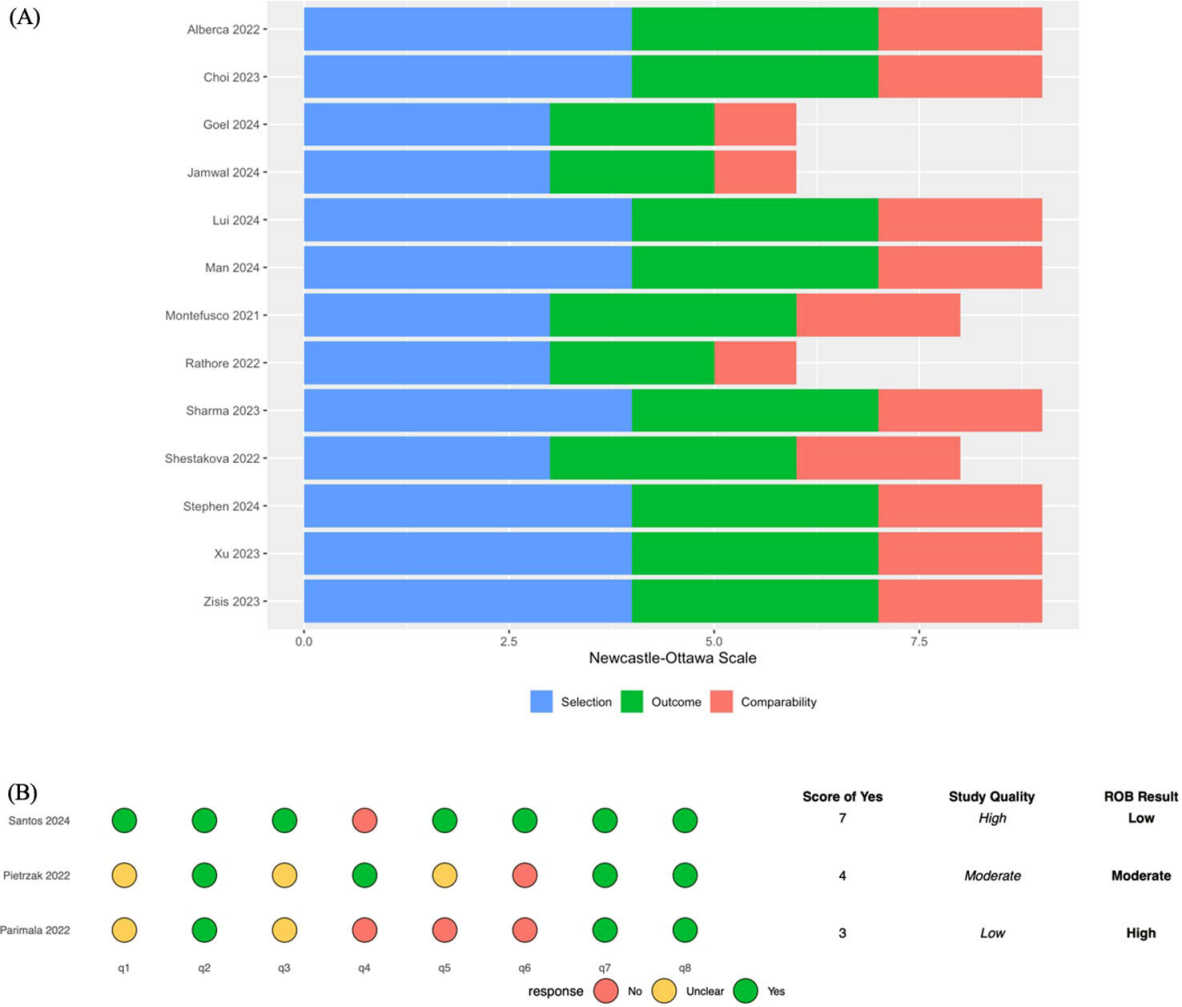
Methodological quality and risk of bias assessment for the included studies. **(A)** methodological quality of cohort studies using the Newcastle-Ottawa scale (NOS). **(B)** quality assessment of cross-sectional studies using the JBI critical appraisal checklist. Green indicates low risk, yellow indicates moderate/unclear risk, and red indicates high risk of bias.

The methodological quality and risk of bias of the included studies are summarized in **Figure 2**. Among the 16 included studies, 13 cohort studies were assessed using the Newcastle-Ottawa Scale (NOS) (**Figure 2A**). Overall, study quality was moderate to high, with NOS scores ranging from 6 to 9. Most studies demonstrated strong performance in the selection and outcome domains, while variability was primarily observed in the comparability domain.

The remaining three cross-sectional studies were evaluated using the Joanna Briggs Institute (JBI) Critical Appraisal Checklist (**Figure 2B**). Santos et al. (24) was classified as high quality (low risk of bias), with a score of 7/8, reflecting adequate identification and management of confounding factors. In contrast, Pietrzak et al. (31) and Parimala et al. (29) were rated as moderate and high risk of bias, respectively (scores of 4/8 and 3/8), primarily due to unclear inclusion criteria, limitations in outcome measurement, and insufficient adjustment for confounders.

### Publication bias

Publication bias was assessed through visual inspection of funnel plots for outcomes with a sufficient number of included studies (**Supplementary S2**). For FBG and HOMA-IR, the funnel plots demonstrated a broadly symmetrical distribution of effect estimates around the pooled mean, suggesting no strong evidence of publication bias.

However, interpretation should be made with caution. The number of studies included in each analysis was limited, reducing the reliability of funnel plot assessment. In particular, for the relative risk (RR) analysis of new-onset diabetes, the small number of contributing studies precluded meaningful evaluation of publication bias. Formal statistical tests for funnel plots asymmetry were not performed due to insufficient study numbers.

### Risk of new-onset diabetes following COVID-19 infection

**Figure 3:**
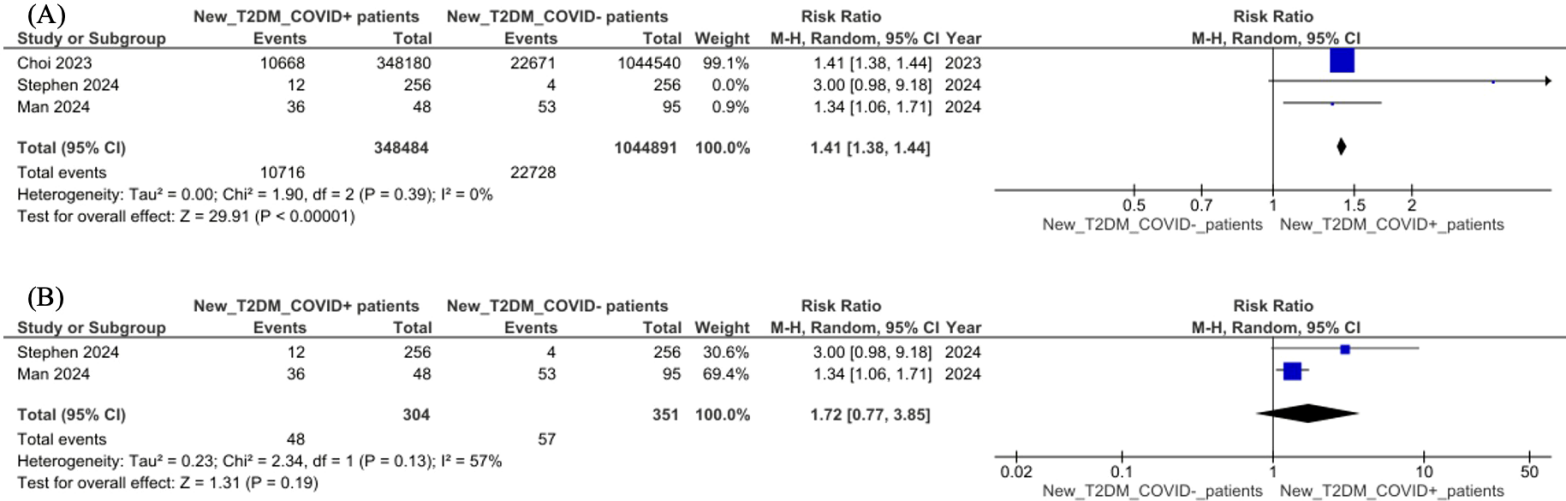
Forest plot of pooled risk ratios (RRs) for new-onset diabetes comparing individuals with prior COVID-19 infection versus non-infected controls. Effect estimates are presented with 95% confidence intervals. **(A)** primary pooled analysis including all studies; **(B)** sensitivity analysis excluding Choi et al. (2023).

Three studies compared the incidence of new-onset diabetes between COVID-19–positive and COVID-19–negative individuals (**Figure 3**). In the primary meta-analysis, the pooled risk ratio (RR) was 1.41 (95% CI: 1.38–1.44; *p* < 0.00001), with no observed heterogeneity (*I²* = 0%). However, this estimate was predominantly driven by a single large nationwide cohort (35), which contributed the majority of the statistical weight.

To assess the robustness of this finding, a leave-one-out sensitivity analysis was performed. After excluding the dominant study, the pooled RR was attenuated to 1.72 (95% CI: 0.77–3.85, *p* = 0.19), with moderate heterogeneity (*I^2^* = 57%). Although the direction of effect remained consistent, the loss of statistical significance indicates substantial uncertainty in the pooled estimate when large-scale studies are excluded.

Taken together, these findings suggest a potential association between COVID-19 and increased risk of new-onset diabetes; however, the strength and precision of this association are currently limited by the small number of studies and the disproportionate influence of a single large dataset. Further well-powered longitudinal studies are required to confirm these findings across diverse populations.

### Fasting blood glucose analysis

Six studies reported FBG values comparing participants with new-onset diabetes to those without (**Supplementary S3**). Individual study estimates showed substantial variability, with some studies reporting higher FBG levels in the new-onset group, while others demonstrated minimal or inverse differences.

The pooled standardized mean difference (SMD) was 0.76 (95% CI: -0.40-1.93; *I^2^* = 97%, *p* = 0.20), indicating no statistically significant difference between groups. However, heterogeneity was considerable (*I^2^* = 97%), suggesting that the observed variability was largely driven by between-study differences rather than chance.

Several factors may contribute to this heterogeneity, including differences in study design, timing of measurement (e.g., acute versus post-acute phase), population characteristics (age, comorbidities, and baseline metabolic status), and variability in glycemic assessment methods. In addition, FBG reflects short-term glycemic status and may be more susceptible to transient physiological fluctuations compared with longer-term markers such as HbA1c.

Given the high heterogeneity and lack of statistical significance, these findings should be interpreted with caution. Overall, the available evidence does not support a consistent difference in FBG between individuals with and without new-onset diabetes following COVID-19.

### Hemoglobin A1c

**Figure 4:**
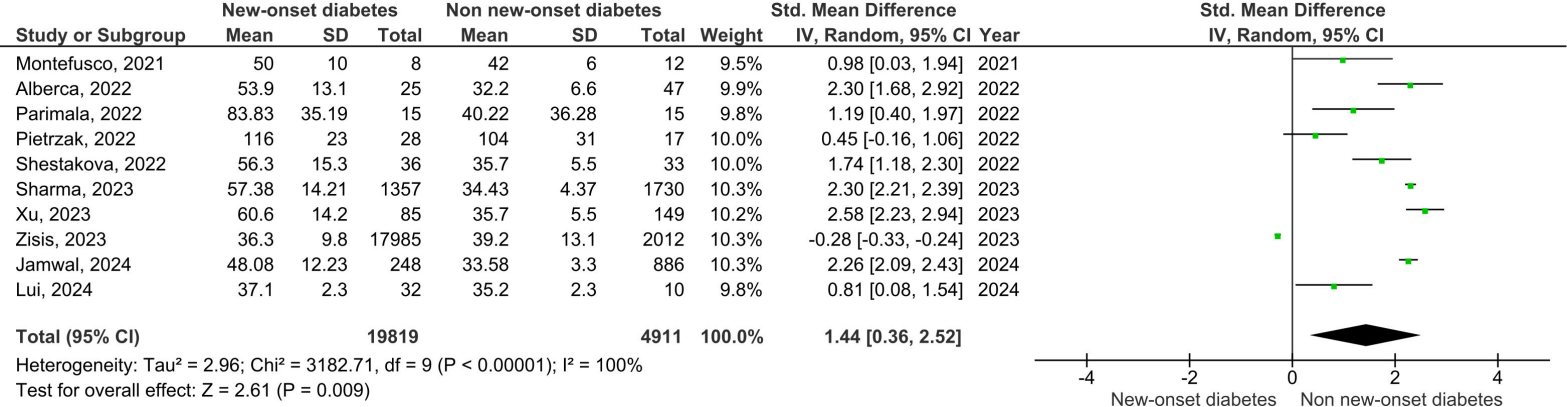
Forest plot of standardized mean differences (SMDs) in HbA1c levels comparing individuals with new-onset diabetes following COVID-19 versus those without new-onset diabetes. Effect estimates are presented with 95% confidence intervals.

Ten studies reported HbA1c levels comparing patients with new-onset diabetes to those without (**Figure 4**). The pooled standardized mean difference (SMD) was 1.44 (95% CI: 0.36–2.52; *p* = 0.009), indicating higher HbA1c levels in individuals with new-onset diabetes following COVID-19.

However, heterogeneity was extremely high (*I²* = 100%), indicating substantial between-study variability. This heterogeneity likely reflects differences in study populations, diagnostic criteria for new-onset diabetes, timing of HbA1c assessment, severity of COVID-19, and treatment or lifestyle factors. Variability in laboratory methods and follow-up duration may also have contributed.

Given this high heterogeneity, the magnitude of the pooled effect should be interpreted with caution. While the direction of association is consistent with impaired long-term glycemic control, the observed effect size may not be directly comparable across studies.

Overall, these findings support an association between COVID-19 and worsening glycemic control among individuals with new-onset diabetes, although further standardized and longitudinal studies are needed to clarify the magnitude and clinical implications.

### Homeostatic model assessment of insulin resistance

Three studies reported data on HOMA-IR values comparing patients with new-onset diabetes to those without (**Supplementary S4**). All included studies demonstrated higher HOMA-IR values in the new-onset diabetes group.

The pooled standardized mean difference (SMD) was 0.96 (95% CI: 0.33–1.58; *p* = 0.003), indicating a statistically significant increase in insulin resistance among individuals with new-onset diabetes following COVID-19. Heterogeneity was moderate (*I²* = 68%), suggesting some variability across studies.

Overall, the findings suggest a consistent signal of increased insulin resistance in post-COVID new-onset diabetes, although further studies are required to confirm the robustness and generalizability of this association (38, 39).

### Unavailable data for pre-specified variables

As specified in the registered PROSPERO protocol, this review aimed to assess changes in FBG, HbA1c, HOMA-IR, insulin levels, and selected metabolites, including dopamine, serotonin, and short-chain fatty acids (SCFAs). However, none of the included studies reported sufficient data on dopamine, serotonin, or SCFAs to permit quantitative synthesis. These outcomes are retained in this report to ensure transparency and adherence to the pre-specified protocol but were excluded from analysis due to the absence of eligible data. No data imputation or substitution was performed.

### Narrative synthesis

**Table 2** summarizes the metabolic and clinical findings of six studies that were not included in the HbA1c meta-analysis. These studies were excluded from quantitative synthesis due to the absence of HbA1c data, use of non-comparable outcome measures (e.g., categorical variables or registry-based diagnoses), or lack of suitable data for effect size calculation. These studies were retained to provide contextual and qualitative insights into post-COVID metabolic outcomes.

**Table 2:** Narrative summary of studies not contributing to the HbA1c meta-analysis (n = 6).

Author (year)	Country/study design	Sample size (N)	Metabolic outcomes assessed	Key findings	Reason not included In Hba1c meta-analysis
Stephen (23)	Nigeria; retrospective cohort	512 (COVID+: 256; Control: 256)	Pre-diabetes and new-onset diabetes prevalence; categorical FBG	Higher prevalence of pre-diabetes (27% vs 4%) and diabetes (7% vs 2%) in COVID+ vs controls	HbA1c not measured; FBG reported categorically only
Man (1)	Romania; prospective cohort	143 (Long COVID: 48; Control: 95)	FBG (~106 mg/dL); HOMA-IR (4.08)	Elevated HOMA-IR indicating insulin resistance in long COVID	HbA1c not reported; contributed to HOMA-IR analysis
Santos (24)	Brazil; cross-sectional	77 (Fatigue: 37; Non-fatigue: 40)	No metabolic biomarkers	No glycemic parameters assessed; focus on fatigue phenotype	No relevant metabolic data available
Zisis (28)	USA; matched nationwide cohort	5,197,096	Incident diabetes (ICD-10 coded)	Increased risk of new-onset diabetes in COVID+ vs matched controls	No biomarker data; contributed to RR analysis
Rathore (30)	India; prospective cohort	100 (Pre-diabetes: 42; Non-diabetes: 58)	FBG (continuous); incident dysglycemia	Higher FBG in those developing NODM/NOPD	HbA1c not measured
Choi (35)	South Korea; nationwide matched cohort	1,392,720	Incident diabetes (registry-based)	Increased risk of new-onset diabetes; dominant contributor to RR analysis	No biomarker data; contributed to RR analysis

FBG, fasting blood glucose; HbA1c, glycated hemoglobin; HOMA-IR, Homeostatic Model Assessment of Insulin Resistance; NODM, new-onset diabetes mellitus; NOPD, new-onset prediabetes; RR, relative risk; SMD, standardized mean difference; COVID+, COVID-19 positive; COVID−, COVID-19 negative; ICD-10, International Classification of Diseases, 10th revision; EHR, electronic health record. † All studies were included in the qualitative synthesis. Zisis (2023) and Choi (2023) contributed to the RR meta-analysis; Man (2024) contributed to the HOMA-IR analysis; Stephen (2024) and Rathore (2022) contributed to descriptive FBG analyses..

## Discussion

This systematic review and meta-analysis suggests an increased risk of new-onset diabetes among individuals recovering from COVID-19 compared with those without prior infection. The primary pooled estimate indicated a higher risk (RR 1.41; 95% CI: 1.38–1.44); however, this finding was predominantly driven by a single large nationwide cohort. Sensitivity analysis excluding this study resulted in loss of statistical significance, indicating that the magnitude and precision of the association remain uncertain. These findings are broadly consistent with large-scale epidemiological analyses reporting elevated post-COVID diabetes risk (10, 11, 40). Taken together, the available evidence supports a potential association between COVID-19 and increased diabetes risk, although the strength and stability of this relationship requires further confirmation.

These findings should also be interpreted in the context of recent systematic reviews and large-scale epidemiological studies. For example, Cocking et al. (2025) reported a similarly increased risk of new-onset diabetes following COVID-19 infection (RR 1.41) based on large cohort data (41). More broadly, prior cohort studies and meta-analyses have consistently suggested an elevated risk of incident diabetes after SARS-CoV-2 infection (10, 11, 42).

While our findings are aligned with this emerging body of evidence, the present study extends the literature in several important ways. First, beyond incidence-based outcomes, we incorporate quantitative synthesis of metabolic biomarkers, including HbA1c and HOMA-IR, providing insight into underlying pathophysiological changes associated with post-COVID metabolic dysfunction. Second, unlike prior meta-analyses that often report prevalence without comparator groups, we evaluate relative risk while explicitly addressing the influence of dominant datasets through sensitivity analysis. Third, by restricting our analysis to the post-acute phase (≥3 months), we better distinguish persistent metabolic dysregulation from transient stress hyperglycemia, a limitation noted in earlier studies with shorter and heterogenous follow-up periods (42).

Methodologically, our findings also highlight the extent to which pooled estimates may be disproportionately driven by large registry-based studies, underscoring the importance of sensitivity analyses and cautious interpretation. Collectively, these features position our results as complementary to existing epidemiological evidence, while providing additional insight into the persistence, heterogeneity, and potential mechanisms of post-COVID metabolic dysregulation.

The mechanisms underlying this association are likely multifactorial and remain incompletely understood. Direct viral effects on pancreatic β-cells, mediated through ACE2 receptor expression, may impair insulin secretion in susceptible individuals. In addition, accumulating evidence suggests that SARS-CoV-2 infection induces persistent immune activation and inflammatory signaling, characterized by elevated cytokines such as IL-6, TNF-α, and IL-1β, which disrupt insulin signaling pathways and promote insulin resistance (11, 43). These inflammatory cascades may impair insulin receptor substrate (IRS) signaling and downstream PI3K/Akt pathways, contributing to reduced glucose uptake and metabolic dysregulation. Systemic responses to infection, including stress-related hyperglycemia and prolonged low-grade inflammation, may further exacerbate these effects (44). The observed elevations in HbA1c (SMD 1.44; 95% CI: 0.36–2.52) and HOMA-IR (SMD 0.96; 95% CI: 0.33–1.58) are consistent with impaired long-term glycemic control and increased insulin resistance, although the magnitude of these effects should be interpreted with caution given substantial heterogeneity.

In contrast, findings for FBG were inconsistent (SMD 0.77; 95% CI: −0.40 to 1.94) and characterized by substantial heterogeneity, in line with prior reports (45, 46). This variability likely reflects differences in study design, timing of follow-up, laboratory methods, patient characteristics, and concurrent treatments such as corticosteroids. Notably, HbA1c and HOMA-IR appeared to provide more stable signals across studies, suggesting that these measures may better capture sustained alterations in glycemic control and insulin sensitivity following COVID-19.

Established cardiometabolic risk factors, including age, obesity, hypertension, cardiovascular disease, and chronic kidney disease, are also associated with increased risk of severe COVID-19, underscoring the broader interplay between metabolic health and infection outcomes (47, 48). Furthermore, severe infection and the use of glucocorticoids may exacerbate hyperglycemia. The inflammatory response to SARS-CoV-2 infection may further impair insulin signaling and β-cell function, reinforcing a cycle of metabolic and immune dysregulation (49). These observations align with the emerging conceptualization of diabetes as an immunometabolic disorder, in which metabolic and immune pathways are tightly interconnected and may be amplified following viral infections (18).

A key strength of this review is the application of design-specific quality assessment tools, the NOS for cohort studies and the JBI checklist for cross-sectional studies, enabling a more tailored evaluation of risk of bias. In addition, the use of a random-effects model provided conservative estimates that account for heterogeneity across populations and study designs.

Several limitations should be considered. Although 16 studies were included, only three contributed to the pooled risk ratio analysis, limiting the robustness of the primary estimate. The dominance of a single large-scale study further constrains interpretability. While funnel plots were examined, assessment of publication bias is limited when fewer than ten studies are available. Substantial heterogeneity was observed across metabolic outcomes, particularly for FBG and HbA1c, likely reflecting differences in population characteristics, disease severity, and timing of biomarker assessment. In addition, several pre-specified metabolites, including dopamine, serotonin, and SCFAs, were not reported in eligible studies, limiting insight into broader mechanistic pathways.

Despite these limitations, the observed pattern of impaired glycemic control and increased insulin resistance following COVID-19 has important clinical implications. Routine metabolic evaluation may be warranted as part of post-COVID care, particularly among individuals with pre-existing cardiometabolic risk factors. From a research perspective, prospective longitudinal studies are needed to clarify the temporal trajectory of metabolic dysfunction and to better quantify risk. Future studies incorporating comprehensive biomarker profiling—including fasting insulin, C-peptide, inflammatory mediators, and microbiome-related metabolites—may help elucidate underlying mechanisms and identify potential therapeutic targets (50).

## Conclusion

This systematic review and meta-analysis suggests that COVID-19 infection is associated with an increased risk of incident diabetes, accompanied by persistent impairments in glycemic control and insulin resistance. The observed elevations in HbA1c and HOMA-IR are consistent with sustained metabolic dysregulation following SARS-CoV-2 infection, potentially driven by a combination of β-cell dysfunction and chronic immune-mediated insulin resistance.

From a clinical perspective, these findings support the integration of metabolic monitoring into post-COVID care pathways, particularly for individuals at elevated cardiometabolic risk. However, given the limited number of studies contributing to pooled risk estimates and the heterogeneity across outcomes, the magnitude of these effects should be interpreted with caution. At a population level, the long-term metabolic consequences of COVID-19 warrant continued surveillance and well-designed longitudinal studies to clarify causal pathways and inform preventive strategies.

## Data Availability

The original contributions presented in the study are included in the article/**Supplementary Material**. Further inquiries can be directed to the corresponding authors.

## Supplementary material

Supplementary S1 Detailed search strategies used for PubMed and Embase databases, including keywords, Boolean operators, and applied filters.

Supplementary S2 Funnel plots of standardized mean differences (SMDs) in (A) FBG and (B) HOMA-IR comparing new-onset versus non-new-onset diabetes groups.

Supplementary S3 Forest plot of standardized mean differences (SMDs) in fasting blood glucose (FBG) comparing new-onset versus non-new-onset diabetes groups.

Supplementary S4 Forest plot of standardized mean differences (SMDs) in HOMA-IR comparing new-onset versus non-new-onset diabetes groups.
