# Supplementary figures and images for: Post-acute metabolic changes and risk of new-onset diabetes following COVID-19: a systematic review and meta-analysis
Source: Front Endocrinol (Lausanne). 2026 May 21;17:1835180. doi: 10.3389/fendo.2026.1835180 (PMC13233281; doi:10.3389/fendo.2026.1835180)

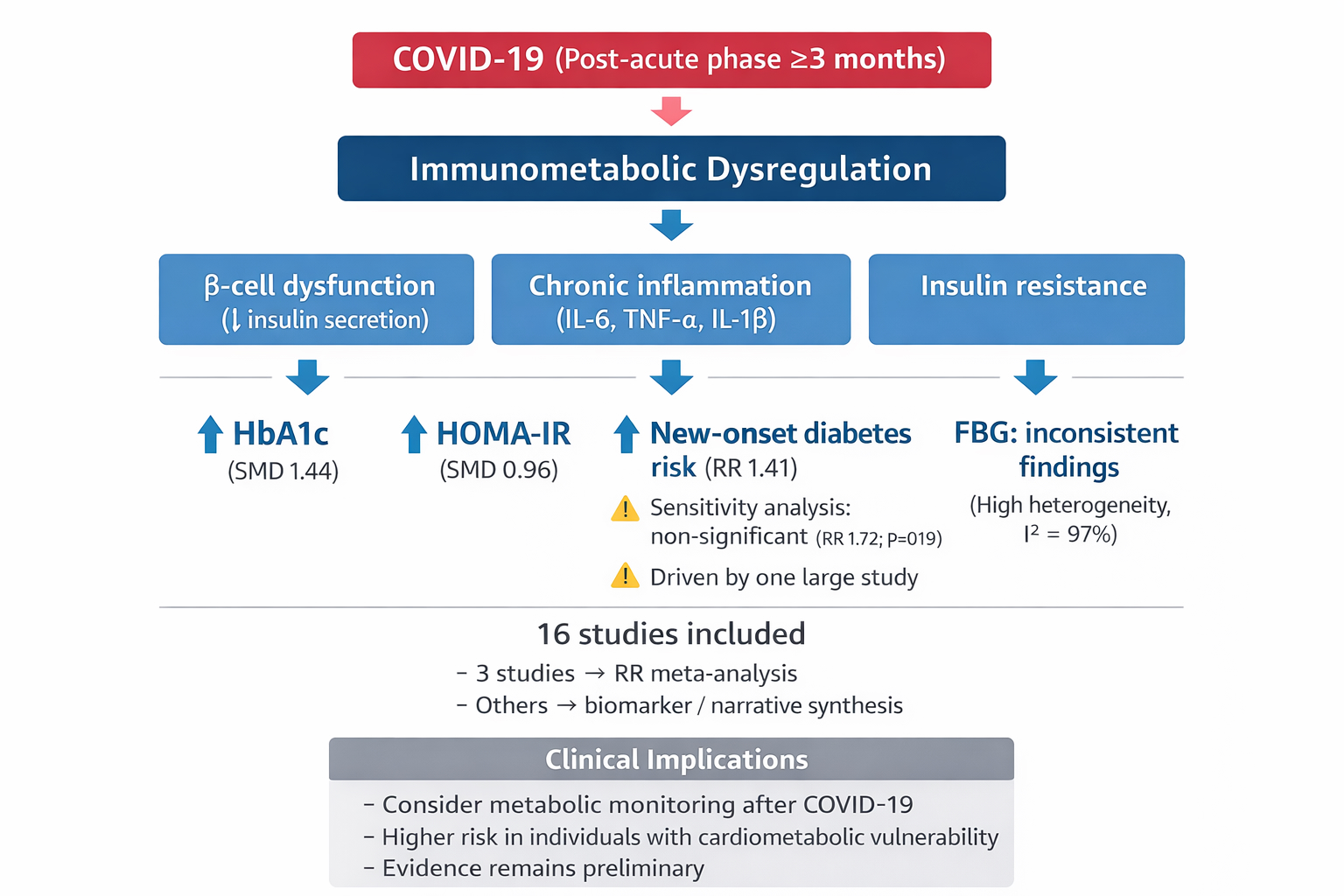

Supplement: Supplementary S1 — Detailed search strategies used for PubMed and Embase databases, including keywords, Boolean operators, and applied filters. [file Image1.jpeg]

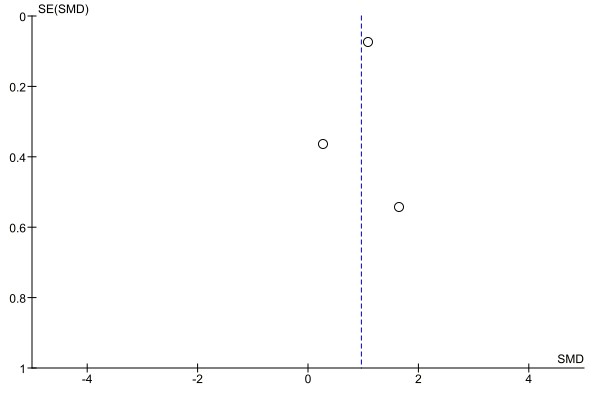

Supplement: Supplementary S2 — Funnel plots of standardized mean differences (SMDs) in (A) FBG and (B) HOMA-IR comparing new-onset versus non-new-onset diabetes groups. [file Image2.jpeg]

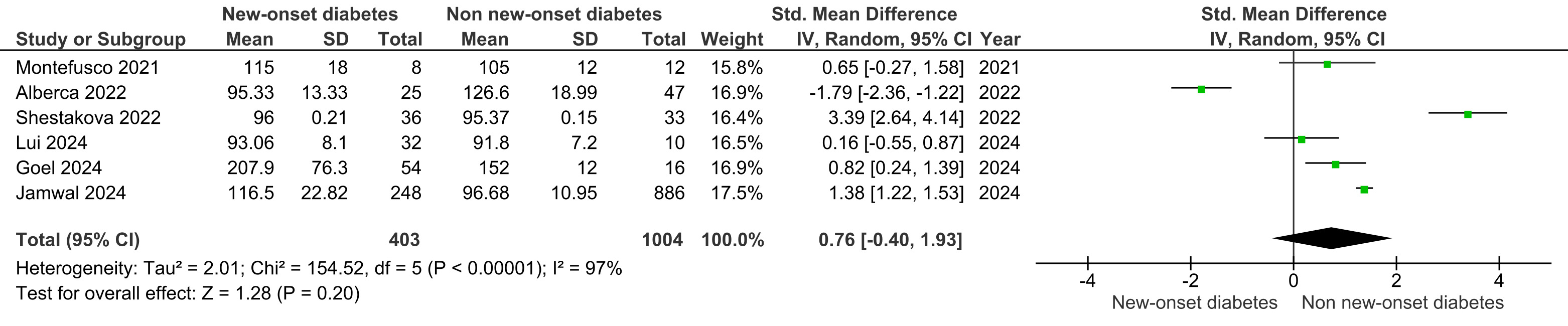

Supplement: Supplementary S3 — Forest plot of standardized mean differences (SMDs) in fasting blood glucose (FBG) comparing new-onset versus non-new-onset diabetes groups. [file Image3.jpeg]

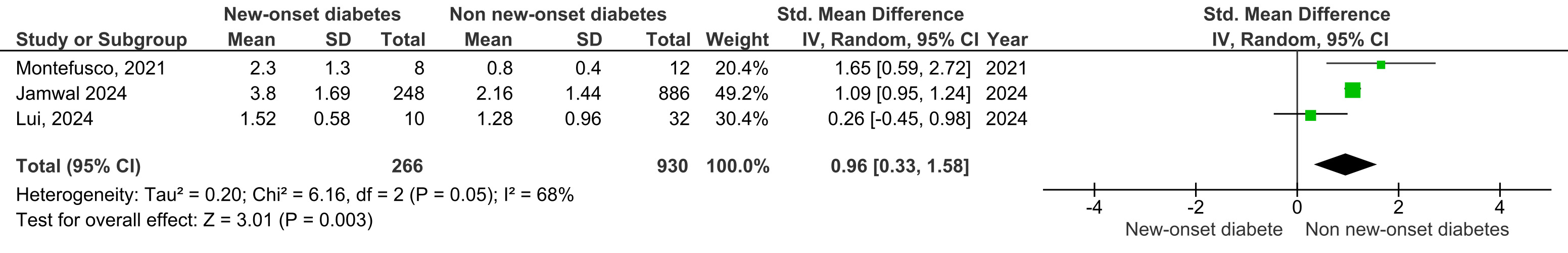

Supplement: Supplementary S4 — Forest plot of standardized mean differences (SMDs) in HOMA-IR comparing new-onset versus non-new-onset diabetes groups. [file Image4.jpeg]
