# Supplementary material for: Post-acute metabolic changes and risk of new-onset diabetes following COVID-19: a systematic review and meta-analysis
Source: Front Endocrinol (Lausanne). 2026 May 21;17:1835180. doi: 10.3389/fendo.2026.1835180 (PMC13233281; doi:10.3389/fendo.2026.1835180)
Supplement: Supplementary file 5 [file Table1.docx]

Supplementary S1. Detailed Search Strategy in PubMed and Embase databases. (Search Date: 18 December 2024)

| Set | Pubmed | Results |
| --- | --- | --- |
| #1 Diabetes | “Diabetes Mellitus”[Mesh] OR “Diet, Diabetic”[Mesh] OR “Blood Glucose”[Mesh] OR “Glucose Intolerance”[Mesh] OR “Insulin Resistance”[Mesh] OR “Insulin”[Mesh] OR diabetes[tiab] OR diabetic*[tiab] OR type2DM[tiab] OR T2DM[tiab] OR prediabetic*[tiab] OR “blood glucose”[tiab] OR “serum glucose”[tiab] OR “blood sugar”[tiab] OR glucosaemia[tiab] OR “plasma glucose”[tiab] OR “serum sugar”[tiab] OR glucosemia[tiab] OR glycaemia[tiab] OR glycemia[tiab] OR glucose intoleranc*[tiab] OR glucose toleranc*[tiab] OR “impaired glucose”[tiab] OR insulin*[tiab] | 1,261,377 |
| #2 Post-Covid-19 | (“post”[tiab] AND (“SARS-CoV-2”[Mesh] OR “sars cov 2”[tiab] OR “covid”[tiab] OR “COVID-19”[Mesh] OR “covid 19”[tiab])) OR “post-acute COVID-19 syndrome”[Mesh] OR (“post acute”[tiab] AND “covid 19”[tiab] AND “syndrome”[tiab]) OR “post-acute COVID-19 syndrome”[tiab] OR (“long”[tiab] AND “covid”[tiab]) OR “long covid”[tiab] | 61,832 |
| #3 Combined | #1 AND #2 | 2,259 |

| Set | Embase | Results |
| --- | --- | --- |
| #1 Diabetes | ‘diabetes mellitus’/exp OR ‘diabetic diet’/exp OR ‘blood glucose’/exp OR ‘glucose intolerance’/exp OR ‘insulin resistance’/exp OR ‘insulin’/exp OR (diabetes OR diabetic* OR type2DM OR T2DM OR prediabetic*):ti,ab,kw OR (‘blood glucose’ OR ‘serum glucose’ OR ‘blood sugar’ OR glucosaemia OR ‘plasma glucose’ OR ‘serum sugar’ OR glucosemia OR glycaemia OR glycemia):ti,ab,kw OR (‘glucose intoleranc*’ OR ‘glucose toleranc*’ OR ‘impaired glucose’):ti,ab,kw OR insulin*:ti,ab,kw | 2,122,159 |
| #2 Post-COVID-19 | ‘long covid’/exp OR ‘post-acute covid-19 syndrome’/exp OR ‘post-acute sequelae of sars-cov-2’:ti,ab OR ‘post-covid’:ti,ab OR ‘post covid’:ti,ab OR ‘long covid’:ti,ab OR ‘long-haul covid’:ti,ab OR ‘long haul covid’:ti,ab OR ‘persistent covid’:ti,ab OR ‘covid-19 sequelae’:ti,ab OR ‘covid-19 survivors’:ti,ab OR ‘sars-cov-2 survivors’:ti,ab | 23,705 |
| #3 Combined | #1 AND #2 | 2,313 |
